# Arabic translation, cultural adaptation, and validation of Australian Pelvic Floor Questionnaire in a Saudi population

**DOI:** 10.1186/s12905-020-01144-w

**Published:** 2021-01-06

**Authors:** Haifaa Malaekah, Haifaa Saud Al Medbel, Sameerah Al Mowallad, Zahra Al Asiri, Alhanouf Albadrani, Hussam Abdullah

**Affiliations:** 1 General Surgery Department, Dr. Soliman Fakeeh Hospital, Fakeeh College of Medical sciences, Jeddah, Kingdom of Saudi Arabia; 2 Princess Nourah Bint Abdulrahman University, Riyadh, Kingdom of Saudi Arabia; 3 Urogynecology and Pelvic Reconstructive Surgery, King Abdullah bin Abdulaziz University Hospital, Princess Nourah Bint Abdulrahman University, Riyadh, Kingdom of Saudi Arabia; 4 Women Health Rehabilitation, King Abdullah Bin Abdulaziz University Hospital, Princess Nourah Bint Abdulrahman University, Riyadh, Kingdom of Saudi Arabia; 5 King Abdullah bin Abdulaziz University Hospital, Princess Nourah Bint Abdulrahman University, Riyadh, Kingdom of Saudi Arabia; 6 General Surgery Resident, King Abdullah bin Abdulaziz University Hospital, Princess Nourah Bint Abdulrahman University, Riyadh, Kingdom of Saudi Arabia

**Keywords:** Arabic, Pelvic floor dysfunction, Questionnaire, Quality of life, Reproducibility of results, Translations

## Abstract

**Introduction and hypothesis:**

The aims of the study were the translation, cultural adaptation, and validation of self-administered Australian Pelvic Floor Questionnaire (APFQ) on a Saudi population.

**Methods:**

The translation and cultural adaptation was performed in 854 women over 18 and not pregnant who agreed to answer the Arabic version of the questionnaire. The content/face validity, internal consistency (reliability), and construct validity (factor analysis) were assessed. Statistical analysis was carried out using SPSS 24.0 statistical software.

**Results:**

The Cronbach’s alpha results were above 0.8 for the questionnaire’s overall reliability (bladder function: 0.877, bowel function: 0.834, prolapse symptoms: 0.784, sexual function: 0.762) showing adequate internal consistency reliability and high statistical significance. A statistically significant correlation was observed among the 40 items of the questionnaire. The issue of multicollinearity was not found, and the determinant of the correlation matrix was 0.001. A value of > 0.5 was achieved when the Kaiser–Meyer–Olkin and Bartlett’s tests measured 0.806 and the Bartlett’s test of sphericity was statistically significant χ^2^ (780) = 4150.46 (*p* < 0.001). The values of loading indicate that all 4 factors (bladder function, bowel function, prolapse symptoms, sexual function) contributed to each of their items.

**Conclusions:**

This study provides the Arabic version of the self-administered APFQ as a reliable and valid instrument for evaluating symptom severity and impact of pelvic floor dysfunction on the quality of life of Arabic women. It also will enable the researchers from Arab countries to use this instrument to assess pelvic floor dysfunction prevalence in their settings.

## Background

Pelvic floor dysfunction (PFD) includes different aspects of symptoms from urinary incontinence (UI), fecal incontinence (FI), Pelvic organ prolapse (POP) to sexual dysfunction, all of which can affect to the quality of life (QOL) and limit social activity [1].

A validated questionnaire plays a fundamental role in identifying symptoms of a disease, helping clinicians in assessing and characterizing any symptom objectively [2]. Consequently, simplified questionnaires for assessing the clinical manifestations and patients’ physical, social, and emotional responses to the disease process should be developed in an effort to help healthcare workers to perform sufficient clinical evaluations [3].

It is estimated that 25% of all women in the United State of America are affected by PFD and almost 20% of these women will need surgery for UI or POP at some period in their lives [4].

The Australian questionnaire is a validated, reliable questionnaire that can be used in clinics to assess all the aspects of PFD’s symptoms, severity, and its impact on QOL in an easy and reliable way. There are many questionnaires available to assess the symptoms of PFD, their severity, and the impact of the symptoms on QOL, yet not all of them assess all the aspects of the disease (bowel, bladder, prolapse, and sexual dysfunction). The Australian PFD questionnaire is the exception, which first came out as an interview-based questionnaire to assess all the symptoms, severity, and QOL of PFD in a reliable and valid way [5]. Later it was validated to be used as a self-administered questionnaire to be used in routine clinics [6].

There is evidence of an increase in the prevalence of UI in the Saudi population compared to the international population (41.4% vs 27.6%) [7]. However, to our knowledge, there is no documented prevalence for other components of PFD in the Saudi population. Hence, there is an immense need for a valid and reliable Arabic version that can assess all components of PFDs. The objective of this study was to translate the Australian Pelvic Floor Questionnaire (APFQ) into Arabic, validation it linguistically, and adapt it culturally, to make the questionnaire useful for the Arabic community and researchers.

## Materials and methods

### Study population

The final Arabic version of the APFQ was tested using 854 female participants. The study subjects were selected randomly and voluntarily. The data was collected through self-administered online surveys, designed using Survey Monkey. Aiming to reach to a large population, we sent the survey to employees at Princess Nourah bint Abdulrahman University, their friends and relatives through emails, Twitter, and WhatsApp. Moreover, we recruited participants from waiting areas at King Abdullah bin Abdulaziz University Hospital through signage. We included women who agreed to participate, were not pregnant, over 18, and literate. The participants were asked about any word they did not understand as well as any word or expression that they found unacceptable or offensive. The misunderstood words or questions were identified, and the recommendations of participants were obtained. Afterward, the needed changes were made to the questionnaire by the researchers, and the Arabic version of the APFQ was then used for validation. A pilot study with 55 subjects was carried out to gauge the feasibility of using the questionnaire by assessing face validity, content validity, and internal consistency.

### Questionnaire description and adaptation

We used an electronically-based, self-administered validated APFQ questionnaire in addition to demographic data and obstetric risk factors [5]. It is a validated questionnaire consisting of 43 questions on the symptoms of PFD. It has 4 domains: bladder function (Q1–15), bowel function (Q16–27), prolapse symptoms (Q28–32), and sexual function (Q33–42).

### Translation process

After obtaining permission from the author, we started the validation process of the APFQ by translating it to Arabic by 2 independent, bilingual, native Arabic speakers. One translator had a medical background and the second translator was an official translator (non-medical background). Then we compared the 2 versions until we reached agreement.

The Arabic version was translated back to an English version by another bilingual Arabic-to-English translator who was a native Arabic speaker. The Arabic version of the questionnaire was compared with the original English version of the questionnaire by the research team.

Then, a meeting was carried out to produce a translated Arabic version after minor linguistic changes. In attendance were 2 translators, the study’s principal investigator, and another person to act as an adjudicator who never saw the survey and had experience in PFD.

### Validation process

*Content/face validity* The questionnaire was assessed with a test group (55 volunteer participants). After filling it out, the participants were interviewed individually or in groups about the questionnaire and discussed the unclear areas. Two PFD experts (HM, SM) then discussed these points with each other, and the final version of the survey was developed (Additional file 1).

*Internal consistency (reliability)* Internal consistency with Cronbach’s alpha, a value of 0.75 was observed with data of the pilot study.

### Ethical issues

Before beginning the study, permission was obtained from the original author (Dr. Caven Baessler) of the English version for the use of the questionnaire. Informed consent was obtained from the study participants. Then, ethical approval was obtained from Princess Nourah bint Abdulrahman University Ethics Committee with decision no: 19-0198.

### Statistical analysis

The data were analyzed using SPSS version 24.0 statistical software (IBM Inc., Chicago IL, USA). Descriptive statistics (mean, standard deviation (SD), frequencies, and percentages) were used to describe the study variables. The internal consistency of the Arabic PFD Questionnaire was assessed using Cronbach’s alpha. Pearson’s correlation coefficient among the items was calculated to evaluate convergent validity of the questionnaire. The construct validity of the questionnaire was determined by using factor analysis, where correlation matrix, Kaiser–Meyer–Olkin (KMO) measurement of sampling adequacy, and Bartlett’s test of sphericity were used to assess the factorability of 40 items. The factor structure was examined by applying the principal component method. The proportion of variance was estimated through initial Eigen values explained by each of the factors. A varimax rotation was used to obtain the rotated factors. A scree plot was used to ascertain the number of factors. A *p* value of ≤ 0.05 was used to report the statistical significance of results.

## Results

Of the 854 female study subjects, 88 (15.3%) were aged 18–29 years, 203 (35.2%) were aged 30–39 years, 187 (32.5%) were aged 40–49, and the remaining were 50 years of age or older. Six hundred fifty three (79.2%) women were Saudi and the remaining was non-Saudi (20.8%). The educational status of college degree or higher was held by 549 (95.3%) subjects and 89.6% were married. More than 60% were overweight or obese.

The mean (SD) of the responses to all items from the 4 domains ranged between a minimum of 0.10 (0.38) for the item “Do you have to push back your prolapse to empty your bowels?” in the “prolapse symptom” domain, and a maximum of 1.98 (0.76) for the item in the sexual function domain “Do you experience pain with sexual intercourse?” The reliability of a questionnaire is the ability to consistently measure an attribute and how well the items fit together conceptually. The internal consistency reliability of each item which was assessed by Cronbach’s alpha where the values ranged between 0.500 and 0.833 (for all the 40 items), that is α value if the item was deleted, which were statistically significant (Table 1).

**Table 1:** Descriptive statistics, correlation, and internal consistency of items if all items in each domain deleted from Arabic Pelvic Floor Dysfunction Questionnaire

Domains and their items	Mean (SD)	Correlated item-total correlation	Cronbach’s alpha if item deleted
*Bladder function items*			
How many times do you pass urine in a day 1	0.36 (0.65)	0.306	0.833
How many times do you get up at night to pass urine 2	0.34 (0.70)	0.341	0.832
Do you wet the bed before you wake up at night 3	0.05 (0.26)	0.206	0.835
Do you need to rush hurry to pass urine when you get the urge 4	0.65 (0.91)	0.476	0.825
Do you leak urine with coughing sneezing laughing or exercising 5	0.51 (0.71)	0.502	0.821
Does urine leak when you rush or hurry to the toilet or can’t you make it in time 6	0.40 (0.65)	0.570	0.817
Do you need to strain to empty your bladder 7	0.23 (0.51)	0.395	0.828
Do you have a feeling of incomplete bladder emptying 8	0.51 (0.72)	0.512	0.820
Is your urinary stream urine flow weak, prolonged, or slow 9	0.28 (0.59)	0.406	0.827
Do you have to wear pads because of urinary leakage 10	0.25 (0.62)	0.515	0.821
Do you limit your fluid intake to decrease urinary leakage 11	0.43 (0.75)	0.476	0.823
Do you have frequent bladder infections 12	0.26 (0.54)	0.425	0.826
Does urine leakage affect activities (recreation, socializing, sleeping, shopping) 13	0.22 (0.57)	0.617	0.815
Do you have pain in your bladder or urethra when you empty your bladder 14	0.22 (0.48)	0.422	0.827
How much does your bladder problem bother you 15	0.51 (0.81)	0.677	0.807
*Bowel function items*			
How often do you usually open your bowels 1	0.23 (0.48)	0.177	0.788
How is the consistency of your usual stool 2	0.23 (0.44)	0.228	0.784
Do you have to strain to empty your bowels 3	0.95 (0.78)	0.619	0.745
Do you use laxatives to empty your bowels 4	0.25 (0.55)	0.400	0.771
Do you feel constipated 5	0.79 (0.70)	0.552	0.755
When you get wind or flatus can you control it or does wind leak 6	0.70 (0.82)	0.335	0.781
Do you get an overwhelming sense of urgency to empty bowels 7	0.66 (0.71)	0.413	0.770
Do you leak watery stool when you don’t mean to 8	0.17 (0.42)	0.289	0.780
Do you leak normal stool when you don’t mean to 9	0.09 (0.41)	0.169	0.788
Do you have a feeling of incomplete bowel emptying 10	0.68 (0.74)	0.651	0.742
Do you use finger pressure to help empty your bowel 11	0.53 (0.78)	0.449	0.766
How much does your bowel problem bother you 12	0.80 (0.88)	0.670	0.736
*Prolapse symptoms items*			
Do you have a sensation of tissue protrusion lump bulging in your vagina 1	0.27 (0.63)	0.594	0.699
Do you experience vaginal pressure or heaviness or a dragging sensation 2	0.26 (0.58)	0.646	0.674
Do you have to push back your prolapse in order to void 3	0.06 (0.28)	0.486	0.752
Do you have to push back your prolapse to empty your bowels 4	0.10 (0.38)	0.433	0.753
How much does your prolapse bother you 5	0.27 (0.63)	0.605	0.694
*Sexual function items*			
Do you have sufficient vaginal lubrication during intercourse 3	0.34 (0.47)	0.314	0.586
During intercourse vaginal sensation is 4	0.58 (0.68)	0.346	0.571
Do you feel that your vagina is too loose or lax 5	1.70 (0.75)	0.084	0.649
Do you feel that your vagina is too tight 6	1.68 (0.85)	0.211	0.619
Do you experience pain with sexual intercourse 7	1.98 (0.76)	0.549	0.500
Where does the pain during intercourse occur 8	0.81 (0.47)	0.444	0.560
Do you leak urine during sexual intercourse 9	1.15 (0.46)	0.191	0.609
How much do these sexual issues bother you 10	0.84 (0.90)	0.471	0.523

The reliability of each of the 4 domains of the questionnaire (0.877, 0.834, 0.784, and 0.762) was found to be highly statistically significant and the overall reliability of the questionnaire (0.888) also shows a high statistical significance (Table 2). The reliability analysis across the 4 age groups in each of the 4 domains shows the Cronbach’s alpha values between 0.80 and 0.85 and not much difference among 4 age groups in any of the 4 domains.

**Table 2:** Reliability (internal consistency) of Arabic Pelvic Floor Dysfunction Questionnaire and its 4 domains

Domains	Cronbach’s alpha (95% confidence interval)	*p* value
All domains	0.877 (0.856, 0.895)	< 0.0001
Bladder function domain	0.834 (0.817, 0.850)	< 0.0001
Bowel function domain	0.784 (0.761, 0.805)	< 0.0001
Prolapse symptoms domain	0.762 (0.736, 0.787)	< 0.0001
Sexual function domain	0.888 (0.871, 0.899)	< 0.0001

**Fig. 1:**
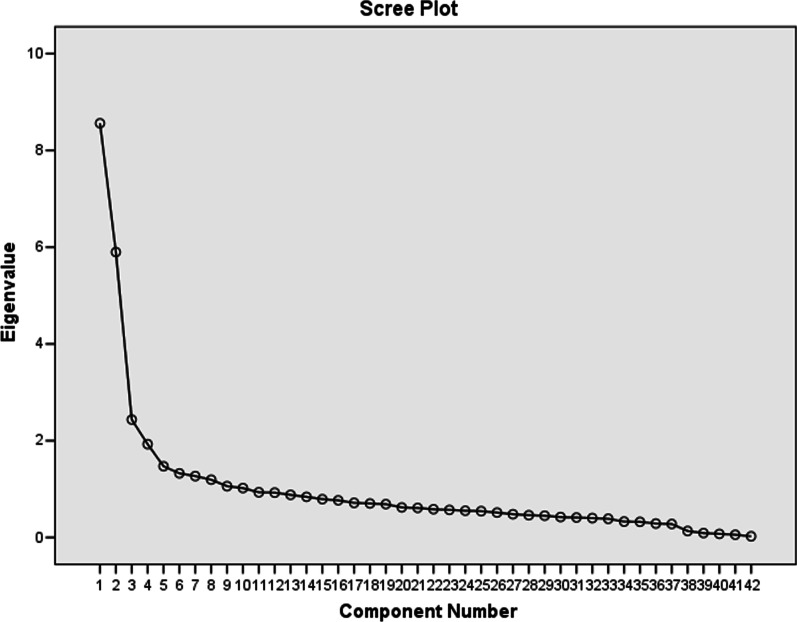
Scree plot of Eigen value and number of factors of Arabic Pelvic Floor Dysfunction Questionnaire

The construct validity of the questionnaire is knows as how much the items of a questionnaire relate to the relevant theoretical construct. It shows the extent to which the intended independent variable (construct) is related to the proxy independent variable (indicator variable). Factor analysis was used to determine construct validity—when an indicator variable consists of multiple items. A statistically significant correlation was observed among the 40 items of the questionnaire. The issue of multicollinearity was not found, and the determinant of the correlation matrix was 0.001, which is greater than the necessary value of 0.00001. That is to say, not only do all 40 items in the Arabic PFD questionnaire correlate well, but none of the correlations are particularly large; hence, none of the 40 items were considered for elimination from the factor analysis. A value of > 0.5 to test the measure of sampling adequacy for factor analysis to continue was achieved when the KMO and Bartlett’s tests measured 0.806 and the Bartlett’s test of sphericity was statistically significant χ^2^ (780) = 4150.46 (*p* < 0.001) which shows that the correlation matrix is not an identity matrix. In addition, the communalities were all greater than 0.45, which indicates that all 45 items were included in the factor analysis. From the factor extraction analysis and with Eigen values, it was observed that the percentage of variance attributed to first factor accounted for 18.6% of the variance, the second factor accounted for 6.79% of the variance, the third factor for 6.15%, and the fourth factor for 5.1% of the variance, which resulted to a cumulative variance of 36.64%. The scree plot is a plot of the Eigen values against all 4 factors. The curve starts to flatten, which occurs after factor number 4 (of x-axis) (Fig. 1). The rotated loadings of the 40 items of Arabic PFD questionnaire on the 4 extracted factors shows that the higher the absolute value of the loading, the more the factor contributes to the variable. The values of loading indicate that all the 4 factors (bladder function, bowel function, prolapse symptoms, and sexual function) contributed to each of their items (Table 3).

**Table 3:** Rotated factor loadings of items from each of the 4 domains of Arabic Pelvic Floor Dysfunction Questionnaire

Domains and their items	Factor number			
	1	2	3	4
*Bladder function items*				
How many times do you pass urine in a day 1	0.404			
How many times do you get up at night to pass urine 2	0.381			
Do you wet the bed before you wake up at night 3	0.411			
Do you need to rush hurry to pass urine when you get the urge 4	0.596			
Do you leak with coughing sneezing laughing or exercising 5	0.581			
Does urine leak when you rush or hurry to the toilet or can’t you make it in time 6	0.635			
Do you need to strain to empty your bladder 7	0.450			
Do you have a feeling of incomplete bladder emptying 8	0.487			
Is your urinary stream urine flow weak, prolonged, or slow 9	0.494			
Do you have to wear pads because of urinary leakage 10	0.608			
Do you limit your fluid intake to decrease urinary leakage 11	0.583			
Do you have frequent bladder infections 12	0.425			
Does urine leakage affect activities recreation, socializing, sleeping, shopping 13	0.741			
Do you have pain in your bladder or urethra when you empty your bladder 14	0.480			
How much does your bladder problem bother you 15	0.695			
*Bowel function items*				
How often do you usually open your bowels 1		0.318		
How is the consistency of your usual stool 2		0.382		
Do you have to strain to empty your bowels 3		0.763		
Do you use laxatives to empty your bowels 4		0.570		
Do you feel constipated 5		0.706		
When you get wind or flatus can you control it or does wind leak 6		0.442		
Do you get an overwhelming sense of urgency to empty bowels 7		0.368		
Do you leak watery stool when you don’t mean to 8		0.495		
Do you leak normal stool when you don’t mean to 9		0.576		
Do you have a feeling of incomplete bowel emptying 10		0.660		
Do you use finger pressure to help empty your bowel 11		0.588		
How much does your bowel problem bother you 12		0.719		
Prolapse symptoms items:				
Do you have a sensation of tissue protrusion lump bulging in your vagina 1			0.582	
Do you experience vaginal pressure or heaviness or a dragging sensation 2			0.511	
Do you have to push back your prolapse in order to void 3			0.631	
Do you have to push back your prolapse to empty your bowels 4			0.719	
How much does your prolapse bother you 5			0.553	
*Sexual function items*				
Do you have sufficient vaginal lubrication during intercourse 3				0.394
During inter course vaginal sensation is 4				0.444
Do you feel that your vagina is too loose or lax 5				0.395
Do you feel that your vagina is too tight 6				0.461
Do you experience pain with sexual intercourse 7				0.771
Where does the pain during intercourse occur 8				0.716
Do you leak urine during sexual intercourse 9				0.558
How much do these sexual issues bother you 10				0.585

## Discussion

There are few validated questionnaires available in the Arabic language. Furthermore, not one of them assesses bladder, bowel, sexual function, and POP symptoms at once. This is the first study that translates the self-administered APFQ from English to Arabic and validates it using an Arabic speaking female study sample. The APFQ evaluates women’s pelvic floor status by questioning bladder function, bowel function, sexual function, and prolapse symptoms all together and as well as measuring the severity of symptoms and their effect on QOL.

Furthermore, other questionnaires only assessed one aspect of PFD. For example, the Arabic Female Sexual Function Index [10] was validated in Arabic and was used only to assess the sexual function just in Arabic speaking females. Moreover, in 2019 Algudairi et al. [11] used the pelvic floor distress inventory (PFDI-20) to evaluate pelvic floor dysfunction in females referred to physiotherapy with chronic back pain [11]. The PFDI-20 comprises 20 items divided into 3 subscales to evaluate distress related to POP, colorectal, and urinary/bladder symptoms. Its main limitation however, was that they did not obtain history of the sexual dysfunction or note if obstetric instruments were used during vaginal delivery, if there were any obstetric injuries, or if episiotomy was performed, which can have major impact contributing to PFD.

We carried out the translation and cultural adaptation of the self-administered APFQ, and then validated it in Arabic speaking women in Riyadh. The questionnaire proved to be reliable, valid, and responsive. The Arabic version of the self-administered APFQ was composed of 43 questions with 4 domains, and comprehensively integrated all areas of pelvic floor disorders including bladder, bowel, prolapse symptoms, and sexual function.

The Pelvic Organ Prolapse/Incontinence Sexual Questionnaire, IUGA-Revised (PISQ-IR) however is validated in Arabic [8], the questionnaire only evaluates the sexual function of women with PFD. An Arabic version of the Global Pelvic Floor Bother Questionnaire was developed and validated by Bazi et al. [9]. It is the only pelvic floor questionnaire that assesses all pelvic floor domains together. It consists of 9 items, but has limited questioning regarding sexuality, as the only question about sexual function is dyspareunia, i.e., pain during sexual intercourse.

For face validity, 55 volunteer participants in our test group filled out the questionnaire; each participant was individually interviewed about the misunderstood words or questions that needed to be clarified. Two pelvic floor experts evaluated the face/content validity. Reliability/internal consistency was evaluated with Cronbach’s alpha and it was adequate as it was above 0.7 in all subscales of the APFQ (Table 2). Our results were similar to the original article in that Cronbach’s alpha for the subscales were as follows: bladder function, 0.83; bowel function, 0.78; sexual function, 0.88; and POP. 0.76 [5].

A limitation of this study is that the self-administered APFQ was only tested in Riyadh, which is the capital city of the Kingdom of Saudi Arabia, so it did not include other areas of the kingdom where cultural differences are huge. Though, Riyadh is a big city where there is a mixture of the population from different regions in KSA settles in Riyadh for purpose of study or work. Future research should focus on expanding different populations and sample size to include other Arab countries too.

The self-administered APFQ was successfully translated and culturally adapted into the Arabic language with the protection of the original meanings of the original English form. The Arabic version of the APFQ is an instrument for evaluation of PFD and it is a useful tool for use in clinical and prevalence studies due to its rapidity and simplicity in being completed and its independence of educational level of the population studied.

## Conclusion

The Arabic version of the self-administered APFQ is a reliable and valid instrument for evaluating symptom severity and impact of PFD on the QOL of Arabic speaking women. It will also enable the researchers from Arab speaking countries to use a well-validated instrument to assess PFD prevalence.

## Supplementary Information

**Additional file 1:** The Saudi Pelvic Floor Questionnaire (SPFQ).

## Data Availability

The datasets used during the current study are available from the corresponding author on upon request.

## Abbreviations

PFD: Pelvic floor dysfunction
QOL: Quality of life
UI: Urinary incontinence
FI: Fecal incontinence
POP: Pelvic organ prolapse
