# Supplementary material for: Arabic translation, cultural adaptation, and validation of Australian Pelvic Floor Questionnaire in a Saudi population
Source: BMC Womens Health. 2021 Jan 6;21:6. doi: 10.1186/s12905-020-01144-w (PMC7789397; doi:10.1186/s12905-020-01144-w)
Supplement: Supplementary file 1 — Additional file 1: The Saudi Pelvic Floor Questionnaire (SPFQ). [file 12905_2020_1144_MOESM1_ESM.pdf]

Prevalence of Pelvic Floor Dysfunction Among Women in Riyadh, Saudi Arabia: A Cross-Sectional Study

You are being asked to voluntarily participate in this survey research study. The purpose of the study is to estimate the prevalence of pelvic floor dysfunction among women in the Riyadh region and the risk factors for it. You are eligible to participate because you are a woman and live in Riyadh. We expect at least 350 will participate in the survey in the Riyadh region.
If you agree to participate, your participation will involve completing a survey. It should take no more than 5 minutes. You may choose not to answer some or all of the questions. Your name will not appear on your completed survey, and no identifying information is being collected as part of this survey.
You may leave the survey at any time before completing it. Whether you complete the survey or not will not affect your health care. There are no known risks from your participation. No direct benefit from your participation is expected. There is no cost to you except for your time. You will not be paid for participation in this study.
Only the study team will have access to the information that you provide, which will remain anonymous. Data from all respondents will be summarized in reports.
You can obtain further information from the principal investigator, Dr. Haifaa Malaekah. If you have questions concerning your rights as a research subject, you may call the PNU Institutional Review Board office at 288-9999 ext. 26913.
Thank you.

Haifaa Malaekah
Consultant Colorectal and General Surgeon
King Abdullah bin Abdulaziz University Hospital
Princess Nourah bint Abdulrahman University
hmmalaekah@pnu.edu.sa

* 1.
Completing this survey indicates your voluntary agreement to participate. By participating in the survey, you are giving permission for the investigator to use your information for research purposes.

- ○ Agree
- ○ Disagree

* 2. Area of residency

- ○ North of Riyadh
- ○ East of Riyadh
- ○ West of Riyadh
- ○ South of Riyadh
- ○ Central Riyadh

* 3. Nationality

- Saudi
- Non Saudi

Please Specify

* 4. Age

* 5. What is your current marital status

- Single
- Married
- Divorcee
- Widow

* 6. Education level

- Primary school and blow
- Intermediate school
- High school
- Post-secondary education (diploma or college degree) or higher

* 7. Occupation

- Student
- non- worker
- worker

Please specify

* 8. Smoking status

- Smoker
- Non-smoker

* 9. Average monthly income level for family

- ◯ Less than 1000 SR
- ◯ 1000 to 4,999 SR
- ◯ 5,000 to 9,999 SR
- ◯ More than 10,000 SR

* 10. Health issues: (you can choose more than one)

- ☐ No
- ☐ Hypertension
- ☐ Heart disease (ex:IHD, arrhythmias)
- ☐ Arthritis
- ☐ Diabetes
- ☐ Bronchial Asthma
- ☐ Multiple sclerosis
- ☐ Stroke
- ☐ Depression
- ☐ Allergy
- ☐ Abdominal surgery
- ☐ Perineal surgery
- ☐ Other, Please Specify

* 11. Weight (kg)

* 12. Height (centimeter)

* 13. Medication history (you can choose more than one):

- ☐ No
- ☐ Non-steroidal Anti-Inflammatory (ex: aspirin, Tylenol, Voltaren, Adol, Panadol, Ibuprofen)
- ☐ Laxative
- ☐ Other, Please specify

* 14. The number of spontaneous vaginal delivery:

* 15. Number of caesarian section (CS);

* 16. History of Instrumental delivery (you can choose more than one answer)

- [ ] Non applicable
- [ ] Forceps Delivery
- [ ] Vacuum Delivery

* 17. Age at first pregnancy (if never get pregnant, choose 0)

* 18. Number of episiotomies

* 19. History of heavy weight lifting

* 20. How many times do you pass urine in a day?

- ○ Up to 7
- ○ Between 8-10
- ○ Between 11-15
- ○ More than 15

* 21. How many times do you get up at night to pass urine?

- ○ 0-1
- ○ 2
- ○ 3
- ○ more than 3 times

* 22. Do you wet the bed before you wake up at night?

- ○ Never
- ○ Occasionally - less than once per week
- ○ Frequently - once or more per week
- ○ Always - every night

* 23. Do you need to rush/hurry to pass urine when you get the urge?

- Can hold on
- Occasionally have to rush–less than once/week
- Frequently have to rush–once or more/week
- Daily

* 24. Does urine leak when you rush or hurry to the toilet or can't you make it in time?

- Not at all
- Occasionally – less than once per week
- Frequently – once or more per week
- Daily

* 25. Do you leak with coughing, sneezing, laughing or exercising?

- Not at all
- Occasionally – less than once per week
- Frequently – once or more per week
- Daily

* 26. Is your urinary stream (urine flow) weak, prolonged or slow?

- Never
- Occasionally – less than once per week
- Frequently – once or more per week
- Daily

* 27. Do you have a feeling of incomplete bladder emptying?

- Never
- Occasionally – less than once per week
- Frequently – once or more per week
- Daily

* 28. Do you need to strain to empty your bladder?

- Never
- Occasionally – less than once per week
- Frequently – once or more per week
- Daily

* 29. Do you have to wear pads because of urinary leakage?

- ○ Never
- ○ As a precaution
- ○ When exercising / during a cold
- ○ Daily

* 30. Do you limit your fluid intake to decrease urinary leakage?

- ○ Never
- ○ Before going out
- ○ Moderately
- ○ Always

* 31. Do you have frequent bladder infections?

- ○ No
- ○ 1-3 per year
- ○ 4-12 per year
- ○ More than one per month

* 32. Do you have pain in your bladder or urethra when you empty your bladder?

- ○ Never
- ○ Occasionally – less than once per week
- ○ Frequently – once or more per week
- ○ Daily

33. Other symptoms (haematuria, pain, etc..)

* 34. Does urine leakage affect your routine activities like recreation, socializing, sleeping, shopping, etc?

- ○ Not at all
- ○ Slightly
- ○ Moderately
- ○ Greatly

* 35. How much does your bladder problem bother you?

- ◯ Not at all
- ◯ Slightly
- ◯ Moderately
- ◯ Greatly

* 36. How often do you usually open your bowels?

- ◯ every other day or daily
- ◯ Less than every 3 days
- ◯ Less than once a week
- ◯ More than once per day

* 37. How is the consistency of your usual stool?

- ◯ Soft
- ◯ Firm
- ◯ Hard (pebbles)
- ◯ Variable
- ◯ Watery

* 38. Do you have to strain to empty your bowels?

- ◯ Never
- ◯ Occasionally – less than once per week
- ◯ Frequently – once or more per week
- ◯ Daily

* 39. Do you use laxatives to empty your bowels?

- ◯ Never
- ◯ Occasionally – less than once per week
- ◯ Frequently – once or more per week
- ◯ Daily

* 40. Do you feel constipated?

- Never
- Occasionally – less than once per week
- Frequently – once or more per week
- Daily

* 41. When you get wind or flatus, can you control it, or does wind leak?

- Never
- Occasionally – less than once per week
- Frequently – once or more per week
- Daily

* 42. Do you get an overwhelming sense of urgency to empty bowels?

- Never
- Occasionally - Less than once per week
- Frequently - once or more per week
- Daily

* 43. Do you leak watery stool when you don't mean to?

- Never
- Occasionally – Less than once per week
- Frequently – once or more per week
- Daily

* 44. Do you leak normal stool when you don't mean to?

- Never
- Always
- Occasionally – Less than once per week
- Frequently – once or more per week
- Daily

* 45. Do you have a feeling of incomplete bowel emptying?

- Never
- Occasionally – Less than one per week
- Frequently – once or more per week
- Daily

* 46. Do you use finger pressure to help empty your bowel?

- Never
- Occasionally – Less than once per week
- Frequently – once or more per week
- Daily

* 47. How much does your bowel problem bother you?

- Not at all
- Slightly
- Moderately
- Greatly

* 48. Do you have a sensation of tissue protrusion/lump/bulging in your vagina?

- Never
- Occasionally – Less than once per week
- Frequently – once or more per week
- Daily

* 49. Do you experience vaginal pressure or heaviness or a dragging sensation?

- Never
- Occasionally – Less than once per week
- Frequently – once or more per week
- Daily

* 50. Do you have to push back your prolapse in order to void?

- Never
- Occasionally – Less than once per week
- Frequently – once or more per week
- Daily

* 51. Do you have to push back your prolapse to empty your bowels?

- ◯ Never
- ◯ Occasionally – Less than once per week
- ◯ Frequently – once or more per week
- ◯ Daily

* 52. How much does your prolapse bother you?

- ◯ Not at all
- ◯ Slightly
- ◯ Moderately
- ◯ Greatly

53. Other Symptoms:(problems: walking/ sitting, pain, vaginal bleeding)

[ ]

* 54. Are you sexually active (if you are single, choose not applicable)?

- ◯ No
- ◯ Less than once per week
- ◯ Once or more per week
- ◯ Daily or most days
- ◯ Not applicable

* 55. If you are not sexually active, please tell us why?

- ◯ Do not have a partner (single, divorce, widow)
- ◯ I am not interested
- ◯ My partner is unable
- ◯ Vaginal dryness
- ◯ Too painful
- ◯ Embarrassment due to the prolapse/incontinence
- ◯ Other (please specify)

[ ]

* 56. Do you have sufficient vaginal lubrication during intercourse?
(if you are single, choose not applicable)

- ◯ Yes
- ◯ No
- ◯ Non applicable

* 57. During intercourse vaginal sensation is:
(if you are single, choose not applicable)

- ◯ Normal/pleasant
- ◯ Minimal
- ◯ Painful
- ◯ None
- ◯ Not applicable

* 58. Do you feel that your vagina is too loose or lax (if you are single, choose not applicable)?

- ◯ Never
- ◯ Occasionally
- ◯ Frequent
- ◯ Always
- ◯ Not applicable

* 59. Do you feel that your vagina is too tight (if you are single, choose not applicable)?

- ◯ Never
- ◯ Occasionally
- ◯ Frequent
- ◯ Always
- ◯ Not applicable

* 60. Do you experience pain with sexual intercourse (if you are single, choose not applicable)?

- ◯ Never
- ◯ Occasionally
- ◯ Frequent
- ◯ Always
- ◯ Not applicable

* 61. Where does the pain during intercourse occur (if you are single, choose not applicable)?

- ◯ Not applicable, I do not have pain
- ◯ At the entrance to the vagina
- ◯ Deep inside, in the pelvis
- ◯ Both at the entrance and in the pelvis
- ◯ Not applicable

* 62. Do you leak urine during sexual intercourse (if you are single, choose not applicable)?

- ◯ Never
- ◯ Occasionally
- ◯ Frequent
- ◯ Always
- ◯ Not applicable

* 63. How much do these sexual issues bother you (if you are single, choose not applicable)?

- ◯ Not applicable
- ◯ Not at all
- ◯ Slightly
- ◯ Moderately
- ◯ Greatly

64. Other symptoms?
(Fecal incontinence, vaginismus, etc.)

[ ]
